# Navigating common pitfalls in metabolite identification and metabolomics bioinformatics

**DOI:** 10.1007/s11306-024-02167-2

**Published:** 2024-09-21

**Authors:** Elva María Novoa-del-Toro, Michael Witting

**Affiliations:** 1 Toxalim (Research Centre in Food Toxicology), Université de Toulouse, INRAE, ENVT, INP- Purpan, UPS, 180 chemin de Tournefeuille St-Martin-du-Touch, BP 3, Toulouse Cedex, 31931 France; 2 Metabolomics and Proteomics Core, Helmholtz Zentrum München, 85764 Neuherberg, Germany; 3 Chair of Analytical Food Chemistry, TUM School of Life Sciences, Technical University of Munich, 85354 Freising-Weihenstephan, Germany

**Keywords:** Metabolomics, Bioinformatics, Mass Spectrometry, Metabolite Identification, Metabolite databases, Data Analysis, LC-MS/MS

## Abstract

**Background:**

Metabolomics, the systematic analysis of small molecules in a given biological system, emerged as a powerful tool for different research questions. Newer, better, and faster methods have increased the coverage of metabolites that can be detected and identified in a shorter amount of time, generating highly dense datasets. While technology for metabolomics is still advancing, another rapidly growing field is metabolomics data analysis including metabolite identification. Within the next years, there will be a high demand for bioinformaticians and data scientists capable of analyzing metabolomics data as well as chemists capable of using in-silico tools for metabolite identification. However, metabolomics is often not included in bioinformatics curricula, nor does analytical chemistry address the challenges associated with advanced in-silico tools.

**Aim of review:**

In this educational review, we briefly summarize some key concepts and pitfalls we have encountered in a collaboration between a bioinformatician (originally not trained for metabolomics) and an analytical chemist. We identified that many misunderstandings arise from differences in knowledge about metabolite annotation and identification, and the proper use of bioinformatics approaches for these tasks. We hope that this article helps other bioinformaticians (as well as other scientists) entering the field of metabolomics bioinformatics, especially for metabolite identification, to quickly learn the necessary concepts for a successful collaboration with analytical chemists.

**Key scientific concepts of review:**

We summarize important concepts related to LC-MS/MS based non-targeted metabolomics and compare them with other data types bioinformaticians are potentially familiar with. Drawing these parallels will help foster the learning of key aspects of metabolomics.

## Introduction

Metabolomics, the systematic measurement of small molecules (< 1500 Da) in a given biological system, represents the newest addition to omics technologies. This approach holds great promise as metabolism is closely linked to the observed phenotype, and metabolomics has shown a tremendous increase in applications over the last few years. Data obtained from the ever-improving analytical methods used in metabolomics are becoming increasingly complex and require the development of more sophisticated data analysis approaches.

Metabolomics works at the interface between biochemistry, analytical chemistry, and bioinformatics and chemoinformatics. Many novel tools for processing and analyzing metabolomics data are published yearly, and there is an increasing demand for scientists capable of understanding and using them. However, metabolites exhibit features distinct from other biological molecules such as DNA, RNA, and proteins. Nowadays, education and training in bioinformatics are mostly centered around these molecules and often overlook the requirements for analyzing small molecules. Different courses try to close this gap, but structured programs are still missing. Therefore, bioinformaticians aiming to enter the field are often overwhelmed by the differences in data structures, requirements, etc.… Individual examples and attempts to close these gaps are created, e.g., the publicly available script “Algorithmic Mass Spectrometry” by Sebastian Böcker (Böcker, 2019). As highlighted in the title of the 2015 review by Johnson et al., “Bioinformatics: The Next Frontier of Metabolomics”(Johnson et al., 2015).

In this article, we do not aim to cover all possible topics of bioinformatics in metabolomics or to comprehensively review available tools; for this, we would like to refer to the excellent reviews conducted by Misra and colleagues (Misra, 2021). Instead, we will discuss the issues and pitfalls we encountered while working together within the framework of the MetClassNet project from 2021 to 2023. In aiming to develop new network-based approaches for analyzing metabolomics data, we began working together and had to learn the scientific terminology of each other’s disciplines. Here, we summarize some pitfalls and concepts that need to be addressed for successful collaboration. We hope that by giving some examples and advice, we can help other bioinformaticians enter the field.

## What can go wrong? Can’t be that hard…

Working with metabolomics data means exposure to different data formats, structures, and problems. We point out four explicit examples that we came across that confused our daily collaboration. Since our project focused on non-targeted metabolomics data obtained by Liquid Chromatography-Mass Spectrometry, including tandem mass spectrometry for generation of fragmentation patterns, (LC-MS/MS), we will also focus on this technique. We suggest several excellent articles and reviews for an overview of LC-MS/MS in metabolomics (Alseekh et al., 2021; Zhou et al., 2012). Here, we focus on a concise description of the data structure. Different software tools for processing LC-MS/MS raw data exist, e.g., xcms, MZmine, MS-DIAL, or commercial solutions from LC-MS/MS vendors or independent suppliers (Benton et al., 2008; Schmid et al., 2023; Tsugawa et al., 2020). Depending on the entry point into a project, a bioinformatician has to deal with raw data and its preprocessing, which includes steps like calibration of the mass-to-charge ratio (*m/z*) axis, peak detection, and chromatographic alignment. Joint work between the authors was based on a so-called feature table and corresponding fragmentation spectra obtained from different LC-MS/MS experiments. This data type was our project entry point and will be the starting point for the following discussion.

The feature table contains multiple columns: *m/z*, retention time (RT), and peak intensities or areas at a minimum. Still, it can also include additional columns, depending on the LC-MS/MS and processing software used, e.g., Collisional Cross Sections (CCS) or peak quality parameters used and exported by the different software tools. The *m/z* and RT pair is typically unique to a feature. Peak intensities or areas represent the quantification value. The higher this value, the higher the concentration of a metabolite. However, these values cannot be directly transferred to concentration values and need calibration to establish a relationship between the concentration and peak intensities/areas. Additionally, this calibration is unique to each metabolite.

**Fig. 1:**
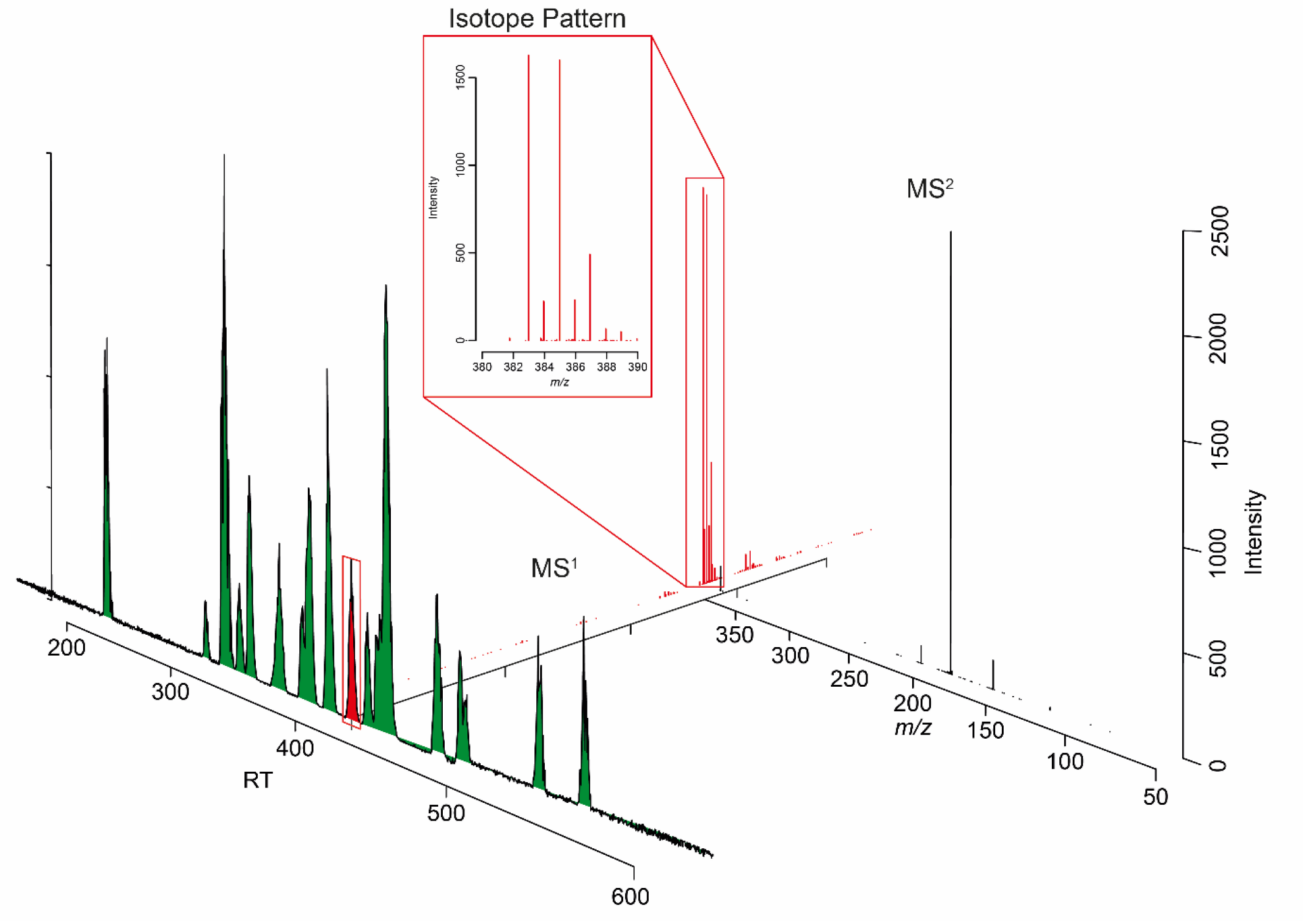
Metabolomics LC-MS/MS data structure. Typical LC-MS/MS data in non-targeted metabolomics consist of a retention time and mass-to-charge dimension (MS^1^) and the associated peak intensity. Additionally, one or multiple MS^2^ spectra might be associated with the detected feature

The peak intensities/areas are typically used for biological analysis using uni- or multi-variate statistics. In the case of data-dependent (DDA) or data-independent acquisition (DIA) modes, features are often associated with fragmentation spectra (see Fig. 1), which are essential for identifying metabolites. It should be noted that not every feature will have a fragmentation spectrum and coverage for current LC-MS/MS instrumentation is somewhere between 30% and 60%. In several cases, laboratories might perform full-scan analysis, meaning only MS^1^ data will be collected during profiling of samples and targeted MS^2^ data will be collected afterwards for features found to be statistically significant.

To facilitate the explanations dedicated to bioinformaticians, we will try to compare metabolomics data to data obtained from, for example, RNA-seq or proteomics (if possible). Additionally, we would like to mention that while LC-MS/MS raw data often uses a standard open data format (.mzML), peak tables from different software tools might look very different. Recent initiatives promoting standardized tabular formats, such as .mzTab, need to be taken up by the software community but are highly welcome (Griss et al., 2014; Hoffmann et al., 2019).

## One metabolite, many, many signals

LC-MS/MS data is complex. A single metabolite can produce multiple signals dependent on its chemical formula and structure. In the ion source of the MS, ions are formed from metabolites and transferred to the gas phase for analysis. Depending on whether the positive or negative ionization mode is used, cationic or ionic pseudo-molecular ions are generated. Certain metabolites will only ionize in one ionization mode, while others can be ionized in both modes. Main adducts are [M + H]^+^ and [M-H]^−^ yielded by adding or subtracting a proton to a neutral molecule M. Though experimentalists aim to generate only a single adduct type, in real life, multiple adducts are formed (Mahieu & Patti, 2017; Nash et al., 2024). Other examples of adducts are [M + Na]^+^, [M + K]^+^, [M + NH_4_]^+^ in positive or [M + FA-H]^−^, [M + HAc-H]^−^ in negative ionization mode. The extent to which the different adducts are observed depends on the structure of the molecule and the experimental conditions, such as the geometry of the ion source, ionization voltages, and the mobile phase composition. The quality of solvents is another critical factor. Water in mass spectrometry quality, stored over a longer period in glass bottles, shows a higher Na and K ions content, which can lead to higher intensities of [M + H]^+^ and [M + K]^+^ adducts (personal observation). On the other hand, ions like [M + NH_4_]^+^ can only be formed if NH_4_^+^ is present in the mobile phase. Certain metabolites require this for ionization, e.g., species from the lipid class triacylglycerols mostly ionize as [M + NH_4_]^+^. Tools are currently being developed to predict adductation and ion species but are far from being used.

In addition to adducts, in-source fragments can also be observed. These happen if conditions in the ion source or any other part of MS before the collision cell lead to fragmentation, primarily due to too high ionization voltages, temperatures, or high voltage drops in ion optics. However, this effect is also dependent on the chemical structure of the metabolite and, consequently, its stability. Therefore, in-source fragmentation is a phenomenon that is not consistent across all metabolites. An example of an in-source fragment is [M-H_2_O + H]^+^ or [M-H_2_O-H]^−^, which can be seen for molecules containing hydroxyl groups. Besides losses of small molecule parts such as hydroxyl, amino, phosphate, or sulfate groups, larger parts can also be lost in in-source fragmentation, e.g., sugar moieties. No general rules for in-source fragments can be stated since they depend on the individual metabolite structures. Sometimes, in-source fragmentation can be so extensive that no intact ion species is observed. For example, the amino acid tryptophan has a very prominent loss of the amino group, leading to an ion with the same *m/z* as indole acrylic acid (*m/z* 188.0706). Under certain conditions, only this ion and no intact [M + H]^+^ are observed.

Additionally, for each observed ion species or adduct, isotopic peaks can be observed, depending on the intensity of the individual adduct, further complicating the spectrum. Most elements have isotopes, which have the same number of protons but different neutrons. Metabolites are often built from carbon (C), hydrogen (H), oxygen (O), nitrogen (N), sulfur (S) and phosphor (P). In nature, the isotope ^13^C is present at 1.1% natural abundance. Therefore, at least one isotopic peak can be observed in most cases. Other isotopes metabolomics are ^15^N (0.4% natural abundance), ^18^O (0.2% natural abundance), and ^34^S (4,37% natural abundance). Beside these, isotopes of chlorine and bromine, potentially found in secondary metabolites or xenobiotics, are of high abundance (^37^Cl (24% natural abundance) and ^81^Br (49.4% natural abundance)).

**Fig. 2:**
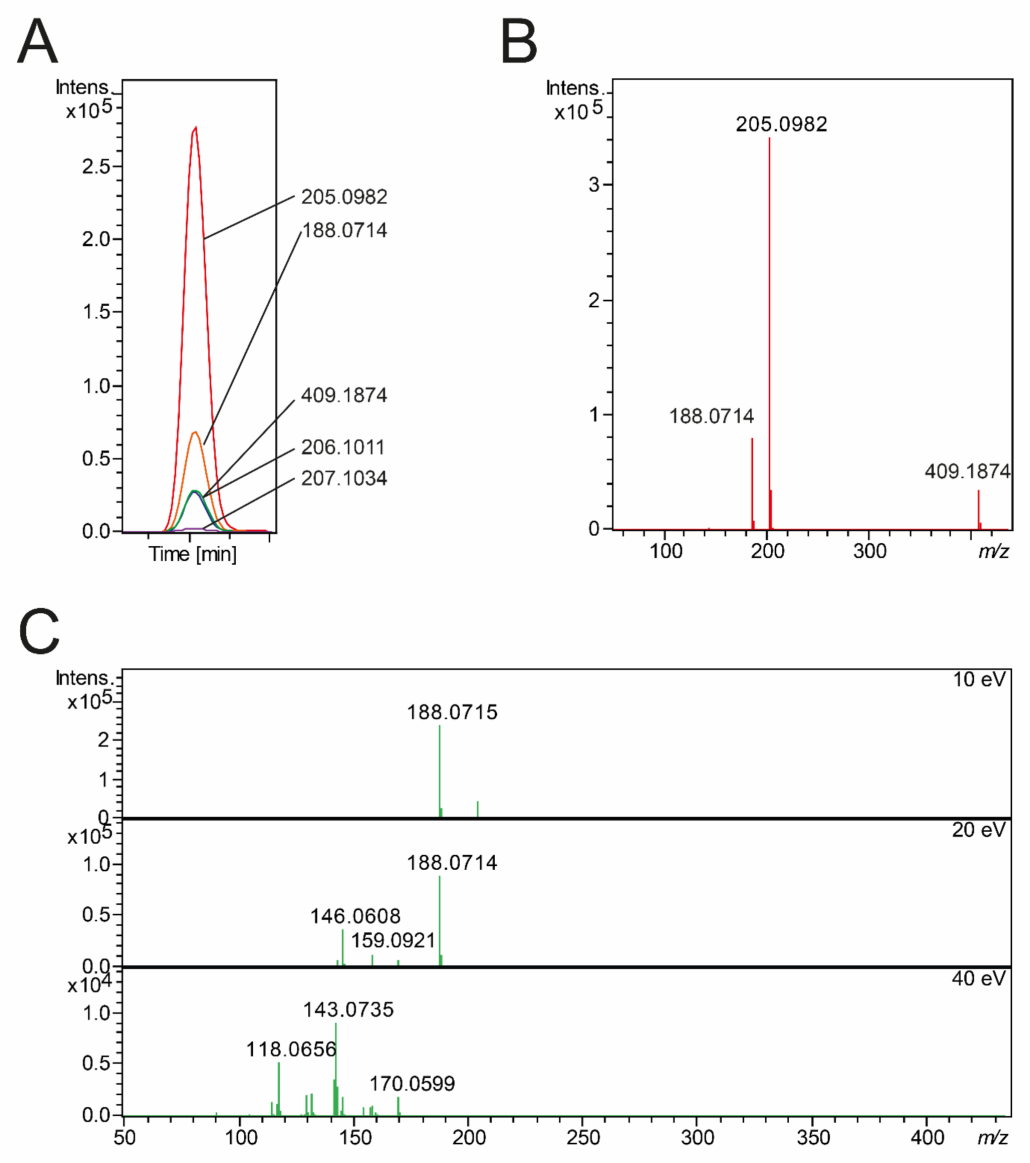
(**A**) Extracted Ion Chromatograms of different m/z derived from tryptophan. m/z 205.0982, 206.1011 and 207.1034 are derived from the [M + H] + adduct and represent the monoisotopic and the first to isotopic peaks. m/z 188.0714 represents an in-source fragment and m/z 409.1874 the [2 M + H]^+^ adduct. (**B**) MS^1^ spectrum of all peaks detected in A. (**C**) MS^2^ spectra of tryptophan collected at different collision energies. All data was generated on a Bruker maXis UHR-TOF-MS

Since isotopes behave chemically (almost) the same and adducts and in-source fragments are formed after the chromatographic separation step in the MS, they will all have the same RT, a fact that can be used for grouping signals. Furthermore, their chromatographic peak shape should be very similar (showing a high correlation across the chromatographic profile). While for isotopes and adducts, defined rules can be used for their identification (e.g., constant distance of [M + H]^+^ and [M + Na]^+^ adducts), this is hardly possible for in-source fragments. Data processing pipelines such as xcms, MZmine, MS-DIAL, or commercial software solutions ideally deal with different adducts, isotopes, and in-source fragments. However, especially in the case of in-source fragments, it cannot be clear if it is an in-source fragment or a molecule with a similar mass and retention time (Domingo-Almenara et al., 2019; Guo et al., 2021). To resolve this ambiguity, metabolite standards are measured to confirm ion species. For example, Fig. 2 shows the MS^1^ spectrum of a tryptophan standard, illustrating different ion species that can be observed. Data were obtained from a Bruker maXis UHR-ToF-MS. For other MS, this spectrum will look different.

While different peaks in the MS^1^ spectrum are informative and allow calculation of the sum formula, they do not allow for derivation of the identity of the metabolite. More detailed information is required in the form of fragmentation spectra to do so. Here, the MS fragments a pseudo-molecular ion into smaller pieces, which might be diagnostic to identify metabolites unequivocally. Differences in the exact MS setup and settings can cause major differences. To lead to fragmentation, external energy must be applied to the ions in the collision cell. This energy is typically referred to as collision energy. Besides collision energy, the actual geometry of the collision cell and the adduct type of the precursor, which shall be fragmented, influence the resulting fragmentation spectrum.

In non-targeted metabolomics, two types of instrumentation are used: Orbitraps and QTOFs. The Orbitrap instrumentation offers two types of fragmentation: CID and HCD, while QTOFs typically offer only CID fragmentation. The difference is that in Orbitraps, the CID is performed in the ion trap, while HCD is performed in the C-Trap. Notably, on Orbitraps, the CID resembles a tandem-in-time configuration type, and the HCD is a tandem-in-space configuration. Each metabolite behaves differently during fragmentation, and the optimal collision energy to obtain the most informative spectrum also changes. However, generic settings are often used since the molecule’s identity is unknown in non-targeted metabolomics. Such generic settings can be suboptimal, leading to under- or over-fragmentation of metabolites that either have too little or too strong fragmentation to be informative. Therefore, multiple collision energies or stepped or ramped collision energies are often used. Figure 2C shows three different fragmentation spectra of the [M + H] + adduct of tryptophan with 10, 20, and 40 eV obtained on a Bruker maXis UHR-ToF-MS. *De novo* identification can often be improved by combining information from multiple collision energies or using ramp spectra (Hoffmann et al., 2022). Different adducts (e.g., [M + H]^+^, [M + Na]^+^ or [M + NH_4_]^+^) will lead to different fragmentation spectra. Besides HCD and CID for Orbitraps and CID for QTOFs as “standard” fragmentation modes, more novel fragmentation methods are on the rise for potential deeper structural insights and enhanced metabolite identification, e.g., Electron Activated Dissociation (EAD), Oxygen Attachment Dissociation (OAD) or Ultraviolet Photodissociation (UVPD).

### Practical implications for the metabolomics bioinformatician

Based on the data and degree of processing performed, the feature table will vary depending on the processing stage. The features can be either ungrouped or grouped only by isotope or by isotope and adduct. It is essential to know the state of the feature table, as specific steps in data processing can be skipped or have to be performed. As a summary for a bioinformatician, each feature in the feature table (i.e., potential metabolite) will be represented as multiple rows, as when various sequences match the same gene during the alignment process of RNA-Seq analysis. Typically, isotopes are first grouped as they are the easiest to detect based on constant mass differences. Most software tools will perform this, or add-on packages exist (e.g., CAMERA for xcms (Kuhl et al., 2011)). Depending on the software or program used, the table either contains rows for isotopes, with a notation denoting them as such, or does not contain isotopic peaks. Further grouping, e.g., of adducts, depends on the software used. Though this adduct grouping is an important step, tools often perform sub-optimally using certain assumptions, e.g., the main adduct is [M + H]^+^, and other adducts can be identified as seed. While this might hold true for many metabolites and grouping might work, unusual adducts or extensive in-source fragmentation might lead to incorrect grouping. In fact, correct adduct annotation can often only be established through metabolite identification (see below). Further software tools for feature table analysis might be based on similar assumptions. To become aware of these, carefully reading the documentation or vignettes associated with software tools will be necessary. Thus, the resulting feature table will need further annotation (i.e., to identify which metabolites you have) to interpret your data. However, as we will explain in the next section, annotation is quite complex.

Apart from the actual feature table, you will likely work with fragmentation spectra and further downstream analysis, such as metabolite identification. It is therefore important to familiarize yourself with software tools or packages that can handle this type of data, e.g., the R package Spectra (Rainer et al., 2022) or the Python package matchms (Huber et al., 2020).

Lastly, remember that the same metabolite measured in different machines and/or using different parameters (e.g., collision energy) will lead to different adducts, in-source fragments, and fragmentation spectra. Therefore, different datasets from different LC-MS/MS instruments will need different parameter settings for processing and feature grouping.

## Metabolite identification is hard!

To correctly identify metabolites, a plethora of information is required, which includes MS^1^ and MS^2^, as well as complementary information, such as retention time (RT) and/or collisional cross sections (CCS). The major problem is that no common unifying physicochemical principle for metabolites (and lipids) can be found in DNA, RNA, or proteins. There is no sequence to which metabolite signals can be mapped. However, the measured features need to be converted to metabolites to correctly interpret non-targeted metabolomics data in a biochemical context.

Different levels of metabolite identification can be achieved (Sumner et al., 2007). One has to differentiate between identification and annotation. While the first one is used when the identity of a metabolite is certain, annotation still gives room for uncertainty. The highest level of identification is achieved by comparing signals from measured biological features against an in-house reference database obtained from chemical reference standards; therefore, identification is limited to the standards available in the laboratory. Matching should be performed based on the exact *m/z*, fragmentation pattern, and RT. For the highest accuracy, matching must be performed using the same collision energies. Lower levels of identification or annotation are achieved by comparing the feature signals against those from public databases, such as GNPS, MassBank, Metlin, etc. Here, the best results are achieved when fragmentation spectra are matched against public spectra from the same instrument and the same or similar collision energies. However, it might be unlikely that precisely the metabolites of interest are available on the employed LC-MS/MS system. Therefore, matching against any available library gives potential ideas about the metabolite identity.

Matching is often limited to MS^1^ data since RTs are commonly not shared or differ too much. Different approaches have been developed to make RT sharing possible and to make it compatible between different laboratories (Aalizadeh et al., 2022; Hao et al., 2023; Renaud et al., 2021; Stoffel et al., 2022). However, coverage of publicly available spectral libraries is limited (Frainay et al., 2018). Fortunately, besides matching against various databases (in-house or public), *in silico* approaches have been developed to increase the coverage of metabolites. In such approaches, structural databases are used instead of spectral libraries. One of the first *in silico* methods developed was MetFrag, which uses combinatorial bond-breaking to find potential fragment structures matching peaks in the fragmentation spectra (Ruttkies et al., 2016). CSI: FingerID, another tool, uses machine learning techniques to learn from fragmentation trees, sum formulas, and molecular fingerprints computed from the fragmentation spectrum to annotate potential compounds (Böcker & Dührkop, 2016; Dührkop et al., 2015). Besides these two, multiple other *in silico* tools exist (Djoumbou-Feunang et al., 2019; Ridder et al., 2014). It is essential to mention that these tools are likely always to give a result, especially if large structural databases such as PubChem are used as input. Therefore, great care needs to be taken when evaluating the results of these tools. The performance of such tools is evaluated in contests like CASMI (CASMI, 2024; Kasama et al., 2014; Schymanski & Neumann, 2013; Shen et al., 2013), for example. However, high scores from *in silico* annotations do not reflect high confidence. A recent method has been established to estimate the confidence of CSI: FingerID results, and it turns out that the performance is similar to library matching (Hoffmann et al., 2022). Though this is a significant step forward, these approaches are still very limited. The use of false discovery rates (FDRs), similar to transcriptomics and proteomics, is still very limited in metabolomics.

Despite the growth of mass spectral libraries and the existence of all these *in silico* tools, annotation and identification often do not achieve the full structural detail as found in metabolite or pathway databases. This is especially true for the position and stereochemistry of double bonds in acyl chains, the position of functional groups (e.g., hydroxyl groups), or stereochemistry in general. For instance, typical RP- or HILIC-MS/MS methods which are employed in non-targeted metabolomics, cannot differentiate between D- or L-Tryptophan, but would require dedicated chiral separation methods (Müller et al., 2014). Fragmentation spectra of both stereoisomers are identical, and retention time cannot be differentiated unless specific chiral chromatography is used. In such cases, one form is often assumed (e.g., the L-form) to be the canonical version in mammals. However, this usually does not hold true when working with bacteria or gut microbiome samples. In the future, pathway analysis tools need to cope with this uncertainty.

### Practical implications for the metabolomics bioinformatician

Best identifications and annotations are obtained using reference libraries measured on the same instrument under the same conditions as the samples of interest. However, the availability of reference standards is limited, and a laboratory is unlikely to hold standards for all metabolites of interest. Significant efforts have been made to increase the number of structures covered in public databases, but directly using a public database would be too easy. In reality, although fragmentation spectra of the same metabolite measured in different machines and/or with different parameters (e.g., collision energy) will generally exhibit an overall similar pattern, they will not be identical. This means that, for the identification to be reliable, the standards should be measured in the same machine (not only one of the same brand and model but literally the same machine) and with the same parameters used to process the samples you are analyzing.

Depending on the organism you are studying, the corresponding metabolites will be known (as when you have a genome of reference in transcriptomics) or not (as in de-novo transcriptome assembly). Even if a metabolite is known, it must have corresponding fragmentation spectra deposited. In the case of databases for metabolite identification and annotation, one needs to differentiate between mass spectral databases holding information on metabolite structures and corresponding tandem MS spectra and metabolite or structural databases containing only structure information. Examples of MS databases are MassBank or GNPS, while KEGG, HMDB, and ChEBI are primary structural databases (though HMDB also contains many spectra from MS and NMR).

Different *in silico* tools such as MetFrag or CSI: FingerID allow the search with MS/MS data in structural databases. Although these tools are improving, their results represent only annotations and not identifications. They need to be treated with care and have to be confirmed by an analytical chemist, ideally using a chemical reference standard. But even with all the tools and databases available, don’t get your hopes up and think that you will annotate every single feature or even most of them, as in RNA-Seq; in most cases, annotation rates in metabolomics are way below 10%, and the remaining 90% are often referred to as the “dark matter” in metabolomics (da Silva et al., 2015).

## Metabolite naming and identifiers are not always unique

The chemical structure of a metabolite is its most unique identifier. Different ways to store the structure electronically exist, e.g., .sdf (structures data file). However, this format is hard to use when working with tabular data. String representations of structures are used in bio- and cheminformatics, e.g., the Simplified Molecular Input Line Entry System (SMILES) or IUPAC International Chemical Identifier (InChI). A hashed version of the InChI, the InChIKey, exists, which is a fixed-length digital representation. Theoretically, the InChIKey is collision-free and, therefore, can serve as a unique identifier. InChIs and InChIKeys can only be generated for molecules with completely known atom connectivity, while SMILES allows for some degree of uncertainty (e.g., exact acyl side chain in a lipid structure). To work with metabolomics and lipidomics results, it is essential to familiarize yourself with the concepts behind SMILES, InChI, and InChIKey, as they are often used in reports. InChIKey can be used to map chemical structures in different datasets, e.g., using only the first of three layers (atom connectivity) with certain restrictions (e.g., all hexoses, such as glucose, fructose, etc…, will have the same first layer). To avoid mismatching due to different representation, it is advised to normalize chemical structures before generating InChIs and InChIKeys, e.g., using the PubChem normalization (Hähnke et al., 2018).

From the structure, the name and chemical formula can be derived. Different names for the same chemicals exist. For example, the chemical IUPAC name of L-Tryptophan is (2 S)-2-amino-3-(1 H-indol-3-yl)propanoic acid. PubChem lists close to 250 synonyms for this single metabolite. Different metabolite databases exist, with the Kyoto Encyclopedia of Genes and Genomes (KEGG) (Sakurai et al.), Chemical Entities of Biological Interest (ChEBI) (Hastings et al., 2016), Human Metabolome Database (HMDB) (Wishart et al., 2021) and Lipid Maps (Liebisch et al., 2020) representing the most used ones. Apart from these, specialized databases that are specific to some organisms exist. For example, SMID-DB stores information on secondary metabolites from the nematode *Caenorhabditis elegans* (Artyukhin et al., 2018). Furthermore, genome-scale metabolic models represent knowledge bases for the metabolism of a given organism. Different tools for analyzing metabolomics results might use different metabolite databases and, therefore, require specific database identifiers. No unified metabolite database as a de-facto standard (similar to UniProt for proteins, for example) currently exists.

Furthermore, the structural details of the identified metabolites might differ from those in the respective databases. For example, while based on standard metabolomics approaches, only tryptophan can be identified (no stereochemistry, see above), the database may contain L- and D-Tryptophan. Additionally, in genome-scale metabolic networks, metabolites might be present at a different charge state, as these models are typically mass- and charge-balanced for microspecies at pH 7.3. ChEBI, for example, stores different versions of metabolites, such as with or without defined stereochemistry or different charge states as separate entities. These individual entities are linked to each other by a rich ontology, and entries in different databases might be connected to each other via cross-references. For example, the KEGG compound C16434, called isoleucine, is linked to ChEBI:38,264, called 2-amino-3-methylpentanoic acid. Though this is correct, as isoleucine is a 2-amino-3-methylpentanoic acid, there also exists a ChEBI:24,898 called isoleucine, which would be the correct link.

### Practical implications for the metabolomics bioinformatician

Individual metabolites might be named by different chemical names or identifiers, which often refer to the same or very similar structure. As a bioinformatician, be aware that not every metabolite database will contain all metabolites; IDs will differ, and direct mapping to databases and metabolic pathways might not be possible due to discrepancies between the level of detail of the identified metabolite and the metabolite in the database/pathway. Different tools for the conversion of identifiers exist and can be incorporated into data analysis pipelines. Depending on the downstream tool used to interpret the results, different identifiers might be required. Therefore, mapping between different databases will be required depending on the results obtained from metabolite identification. Due to the large number of possible names, it is very hard to create automatic workflows to map chemical names. It is advisable to use dictionaries, established databases, and mapping tools. Such mapping can be done with different tools, such as the Chemical Translation Service (CTS) (Wohlgemuth et al., 2010), RefMet (Fahy & Subramaniam, 2020), BridgeDB (van Iersel et al., 2010), and UniChem (Chambers et al., 2013). However, not all metabolites might be present in all databases, which has particular implications, for example, for overrepresentation analysis (see below). In order to generate reproducible results, it is important to work with accurate lists of metabolites present in organisms (e.g., from genome-scale metabolic models) and prepare tables with identifiers, names, etc., in advance. Likewise, similar tables shall be provided by the analytical scientists for the metabolites identified to avoid tedious formatting and conversion. In case of ambiguities, a biochemist and/or an analytical chemist can help identify the most probable match.

Mapping between databases or datasets can be performed using the InChIKey or at least the first block (atom connectivity) to account for potential differences in stereochemistry and charge states. Furthermore, the ontology established by ChEBI can be used for mapping (Poupin et al., 2020). This mapping approach can account for certain ambiguities and was used for example, to map lipids to genome-scale metabolic models. A distance measure is reported based on the distance in the ChEBI ontology. However, this approach is only suitable for entries well established in the ontology, and mapping distances can only be partially used as a quantitative metric.

## Metabolite coverage is low

Typical non-targeted metabolomics can detect several hundred to thousands of features, but only a few hundred metabolites or fewer can be annotated or identified, and even fewer can be detected at the highest confidence level (confirmed by a chemical reference standard). Metabolites span an extensive range of polarity and concentrations. Currently, no single analytical method can cover the entire metabolome. For example, reversed-phase LC-MS/MS (RP-MS/MS) can cover non-polar metabolites such as fatty acids, acyl-carnitines, or bile acids, or even complex lipids, Hydrophilic Liquid Interaction Chromatography (HILIC-MS/MS covers polar metabolites such as amino acids, amines, sugars, and others. Therefore, depending on the employed methodology, only a small subfraction might be sampled and analyzed. To achieve greater coverage, multiple methods need to be combined.

**Fig. 3:**
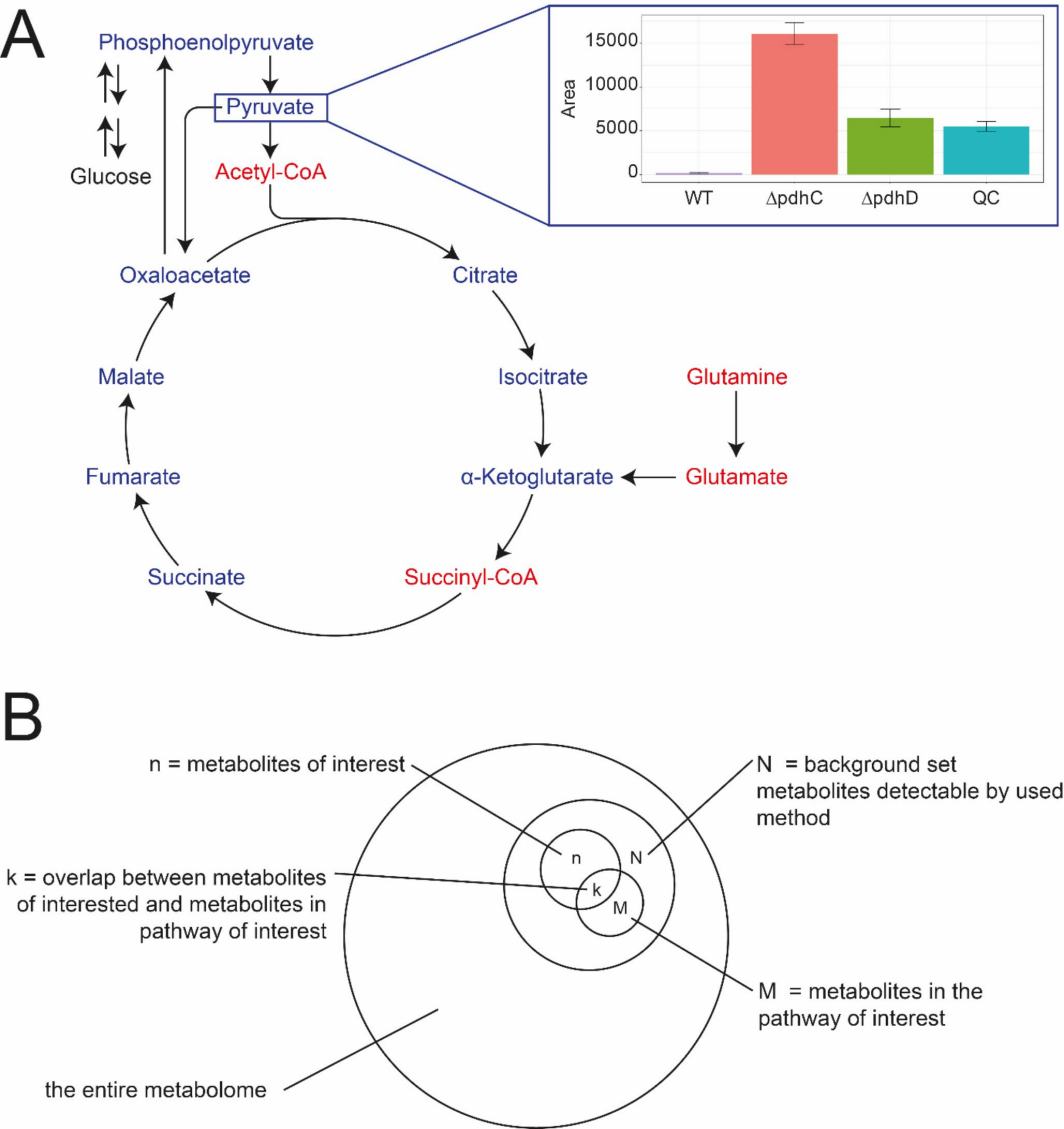
(**A**) Detection of metabolite depends on their physicochemical properties. Certain metabolites can be only detected in negative mode (blue), while other are preferentially detected in positive ionization mode. However, even if a metabolite is theoretically detectable, it might be not present in samples. The example shows relative levels of pyruvic acid in Bacillus subtilis and can be only detected if the downstream consuming enzyme is mutated. (**B**) Metabolite coverage is important for analysis methods such as overrepresentation analysis. The entire metabolome cannot be covered and typically only a subset N is detectable by a specific method. This subset represents the background set for overrepresentation analysis. Figure adopted from Wiederer et al

In many cases, positive and negative ionization modes must be combined to detect sufficient metabolites. For example, single reactions can change the properties quite substantially. For example, the metabolites glutamine, glutamic acid, and 2-oxo-glutaric acid are connected by a linear chain of reactions that feeds into the TCA cycle. However, their physicochemical properties differ. While all of them can be detected in negative ionization mode, only glutamic acid and glutamine are typically detected in positive ionization mode (see Fig. 3A).

Additionally, the metabolome is highly dynamic. Even if metabolites are known and in theory, can be detected by the employed methods (e.g., by measuring a chemical reference standard), they might not be detected because they are either not produced under the given biological conditions or metabolized too fast. Specific metabolites have very high turnover rates and might not be detected when the overall turnover in the reaction cascade is very high. However, the exact flux and turnover are dependent on many different factors, such as growth conditions and nutrient availability. For example, pyruvic acid, an important intermediate between glycolysis and the TCA cycle, can only be detected in *Bacillus subtilis* if the downstream-consuming enzyme (pyruvate dehydrogenase) is mutated, since the flux into further downstream pathways is disturbed and the metabolite accumulates (see Fig. 3A). Furthermore, specific metabolites might not be stable enough to be detected or yield reliable read-outs, including central metabolites such as ATP, NADH or NADPH. Similarly, some metabolites may be present at very low concentrations. The metabolome can span several orders of magnitude in concentration (Bennett et al., 2009). For very low-concentration metabolites, specialized assays with dedicated sample preparation (e.g., solid-phase extraction) and targeted analysis using triple quadrupole MS are often required, rendering them undetectable in non-targeted metabolomics settings. Lastly, for almost all organisms, the complete metabolome remains unknown, so the actual coverage of a method cannot be precisely calculated, which also has implications for the downstream interpretation of results.

### Practical implications for the metabolomics bioinformatician

While transcriptomics or proteomics often produce a single data table for analysis with sufficient coverage of several hundred to thousands of transcripts or proteins, metabolomics often yields multiple feature tables. Depending on the study, these feature tables might need to be joined or analyzed separately. One example is the same chromatographic method’s positive and negative ionization mode. Running multiple methods may be necessary to achieve sufficient coverage of metabolites. Often, this approach is also the case in targeted metabolomics. For example, even though only a single data table is reported, the actual measurement for the commercial Biocrates MxP Quant 500 kit utilizes four different methods. The quality and coverage of metabolomics measurements significantly influence the downstream data analysis and interpretation.

Pathway analysis is one of the major data analysis techniques for the interpretation of metabolomics data, mainly in the form of enrichment or overrepresentation analysis. In contrast to such analyses used in transcriptomics and proteomics, pathway analysis in metabolomics requires great care. The detection of metabolites can be highly biased by the employed analytical method. Therefore, selecting the correct background dataset is important. While the number of detected features is typically very large for transcriptomics and proteomics, and the entire genome or proteome can be used as a background set, this often is not the case in metabolomics. Recently, Wieder et al. published recommendations for the overrepresentation analysis in metabolomics (Wieder et al., 2021). These recommendations included selecting the background dataset, pathway database, and addressing potential misidentifications. To overcome this limitation in coverage, methods for integrating multiple datasets from different methods will be required. While this integration can be achieved for targeted metabolomics and known metabolites, e.g., by metrics established by Boccard et al., similar integrations of multiple datasets of unknown metabolites remain complicated (Boccard & Rudaz, 2018).

Since the coverage of metabolic pathways is often limited by the number of identified metabolites, alternative analysis methods are required. Network analysis has emerged as a suitable alternative to overcome the limitations of pathway analysis. Different types of networks provide different views of biological aspects and can even be integrated with each other (Amara et al., 2022; Salzer et al., 2023).

## Conclusion

Compared to genomics, transcriptomics, and proteomics, metabolomics, and lipidomics are missing one common part: sequences that can be matched against each other. These sequences make it possible to map reads in RNA-seq to the respective genes or lead to specific fragmentation of peptide backbones in peptides, allowing for their sequencing. Furthermore, false discovery rates can be calculated for them, allowing one to judge the goodness of the sequencing result and mapping. Metabolites have no common characteristics that can be used in a similar fashion, making metabolite analysis seemingly complicated from a bioinformatic point of view.

There is a strong need for improved data analytical and bioinformatic tools in metabolomics. These include steps such as raw data processing, metabolite identification, and data interpretation. Though we highlighted several pitfalls and problems in this review, there is great progress in the development of new tools.

In our opinion, teaching in bioinformatics is mostly sequence-based and covers genomics, transcriptomics, and proteomics. Small molecules such as metabolites and lipids, are often not covered or are covered only superficially. Since this is an upcoming topic and more and more metabolomics data will be generated, structured training is required. In this article, only the view of the bioinformatician on specific aspects are covered. However, the other side also needs to adapt. Training for analytical chemists or biochemists needs to involve more basic data science and bioinformatics skills. Data generated by the latest state-of-the-art instrumentation is very dense and requires advanced skills. Despite major challenges still existing, there are many useful resources, protocol papers and guides (Amara et al., 2022; Aron et al., 2020; Heuckeroth et al., 2024; Pakkir et al., 2023). It is important to note that many of these articles and tutorials have been written by analytical chemists and bioinformaticians in collaboration with exactly the goal of bringing together the two fields. Though it seems from this review that often there are more problems than solutions, the authors always found a way to improve their collaboration and overcome all hurdles.

Here are some necessary steps as a summary for (future) metabolomics bioinformaticians to avoid the same pitfalls as the authors:

1. Team up with the (bio)chemist who generated the data as early as possible (best before the data has been generated); it will make your life much easier. Ask if you can join/watch the measurements. Ask many, many questions about the data.
2. Start by figuring out the stage the feature table is in because the steps to follow will depend on this. Examples of useful questions to ask are “Is the feature table grouped or ungrouped?” If it is grouped, “What was used for the grouping: only isotopes or isotopes and adducts?“.
3. Remember that it is common to have multiple rows representing the same feature, and even if your feature table is grouped, the grouping might be incorrect (e.g., due to extensive in-source fragmentation). Additionally, when working with multiple datasets, keep in mind that measurements performed in different machines and/or using different sets of parameters lead to different features. This is relevant for processing and feature grouping (your parameter settings should be adapted for each dataset) and for the annotation/identification step.
4. When annotating your features, keep in mind the difference between identification (knowing the metabolite with certainty) and annotation (finding the metabolite that is likely to represent the feature). You might as well check out some MS databases, such as MassBank and GNPS, as well as tools like MetFrag or CSI: FingerID for *in silico* annotation. Please remember that ~ 90% of the features are expected to remain “unknown”; i.e., you’ll be able to annotate less than 10% of the features.
5. Be aware that the metabolite databases are likely to be incomplete. To complicate things further, the same metabolite can have different names and/or identifiers depending on the database, which does not exactly help when mapping between databases. We, therefore, recommend using predefined dictionaries, established databases, and mapping tools (such as CTS, RefMet, BridgeDB, and UniChem). Candidates to perform such mapping are the InChiKey and the ChEBI.
6. Familiarize yourself with the type of data you’ll need to process (for instance, the feature table and the fragmentation spectra of some or all of the features) and the tools you will need to process it, for example, R packages such as xcms and Spectra are very likely to be useful.
7. By this point, you should already be used to dealing with challenges and uncertainty. Things won’t get any easier for the interpretation step (e.g., via enrichment or overrepresentation analysis). Still, you can take into account the recommendations from Wieder et al. [35] and the metrics established by Bocard et al. [36]. Finally, you can also consider a network approach to interpret your results. As we explored in the MetClassNet project, different networks can be integrated to provide various biological perspectives on the same dataset.

Last but not least, metabolomics data analysis is fun and can be very rewarding once you accept the points above. Increasing the coverage by combining methods, creating better and faster methods, and improving metabolite identification rates are current fields of research in the metabolomics community and are expected to improve significantly in the coming years. There are still many pitfalls, but so far it has been successful, and the field is growing with new solutions and tools being released and published every year.

## Data Availability

No datasets were generated or analysed during the current study.
